# Timing of Loop Ileostomy Closure Does Not Play a Pivotal Role in Terms of Complications—Results of the Liquidation of iLEOstomy (LILEO) Study

**DOI:** 10.3390/jpm14090934

**Published:** 2024-08-31

**Authors:** Michał Kisielewski, Magdalena Pisarska-Adamczyk, Natalia Dowgiałło-Gornowicz, Łukasz Nawacki, Wojciech Serednicki, Mateusz Wierdak, Jerzy Wilczek, Kamil Safiejko, Marcin Juchimiuk, Marian Domurat, Jacek Pierko, Mateusz Mucha, Wojciech Fiedorowicz, Michał Wysocki, Maurycy Ladziński, Michał Zdrojewski, Tomasz Sachańbiński, Tomasz Wojewoda, Victoria Chochla, Karol Tkaczyński, Michał Jankowski, Wojciech M. Wysocki, LILEO Study Group

**Affiliations:** 1 Chair of Surgery of the Faculty of Medicine and Health Sciences, Andrzej Frycz Modrzewski University, 30-705 Krakow, Polandwwysocki@mp.pl (W.M.W.); 2 Department of General and Oncological Surgery, 5th Military Clinical Hospital, 30-901 Krakow, Poland; 3 Department of Medical Education, Jagiellonian University Medical College, 30-688 Krakow, Poland; 4 Department of General, Minimally Invasive and Elderly Surgery, University of Warmia and Mazury in Olsztyn, 10-719 Olsztyn, Poland; 5 Collegium Medicum, Jan Kochanowski University, 25-317 Kielce, Poland; lukasznawacki@gmail.com; 6 2nd Department of General Surgery, Jagiellonian University, 30-688 Krakow, Poland; 7 Department of Oncological Surgery, Specialist Hospital in Brzozow, 36-200 Brzozow, Poland; 8 Colorectal Cancer Unit, Maria Skłodowska-Curie Białystok Oncology Center, 15-027 Białystok, Polandmdomurat@onkologia.bialystok.pl (M.D.); wfiedorowicz@onkologia.bialystok.pl (W.F.); 9 Department of General Surgery and Surgical Oncology, Ludwik Rydygier Memorial Hospital in Krakow, 31-826 Krakow, Poland; 10 “Pro-Medica” Hospital, 19-300 Elk, Poland; 11 Oncological Surgery Clinic, MSWiA Hospital, 10-228 Olsztyn, Poland; mzdrojewski@onet.eu; 12 Oncological Surgery Department with a Sub-Department of Breast Diseases, Tadeusz Koszarowski Oncology Centre in Opole, 45-061 Opole, Poland; saszkin73@gmail.com; 13 Institute of Medical Sciences, Faculty of Medicine, University of Opole, 45-040 Opole, Poland; 14 Department of Oncological Surgery, 5th Military Clinical Hospital, 30-901 Krakow, Poland; 15 Department of Surgical Oncology, Oncology Center, Prof. Franciszek Łukaszczyk Memorial Hospital, 85-796 Bydgoszcz, Poland; karoltkaczynski@gmail.com (K.T.);; 16 National Institute of Oncology, Maria Skłodowska-Curie Memorial, 02-781 Warsaw, Poland

**Keywords:** loop ileostomy reversal, postoperative complications, colorectal surgery, prevention of complications, gastrointestinal surgery

## Abstract

Loop ileostomy is commonly performed by colorectal and general surgeons to protect newly created large bowel anastomoses. The optimal timing for ileostomy closure remains debatable. Defining the timing associated with the best postoperative outcomes can significantly improve the clinical results for patients undergoing ileostomy closure. The LILEO study was a prospective multicenter cohort study conducted in Poland from October 2022 to December 2023. Full data analysis involved 159 patients from 19 surgical centers. Patients were categorized based on the timing of ileostomy reversal: early (<4 months), standard (4–6 months), and delayed (>6 months). Data on demographics, clinical characteristics, and perioperative outcomes were analyzed for each group separately and compared. No significant differences were observed in length of hospital stay (*p* = 0.22), overall postoperative complications (*p* = 0.43), or 30-day reoperation rates (*p* = 0.28) across the three groups. Additional analysis of Clavien–Dindo complication grades was performed and did not show significant differences in complication severity (*p* = 0.95), indicating that the timing of ileostomy closure does not significantly impact perioperative complications or hospital stay. Decisions on ileostomy reversal timing should be personalized and should consider individual clinical factors, including the type of adjuvant oncological treatment and the preventive measures performed for common postoperative complications.

## 1. Introduction

Loop ileostomy is commonly performed by colorectal and general surgeons to protect newly created large bowel anastomoses [1]. Due to the diversion of fecal contents from the large bowel in the event of anastomotic leakage, patients have a lesser risk of major postoperative complications, like fecal peritonitis and potential septic shock. Such complications can have detrimental effects on patients’ short- and long-term operative outcomes [2]. The creation of an independent diverting ileostomy can also negatively affect patient health by leading to electrolyte disturbances, and, in the worst case, to renal insufficiency. Readmissions with acute renal insufficiency increase tenfold in patients with an ileostomy that has not been reversed [3]. Furthermore, ileostomy closure is a separate surgical procedure that can also result in postoperative complications in up to 37% of cases [4]. The standard time of ileostomy closure and restoration of the gastrointestinal tract continuity is around 3 to 6 months [5]. Some surgeons tend to close the ileostomy very early, for example in less than 2 weeks, minimizing the possibility of delayed detrimental effects of the stoma on patients’ physical and mental health. This may not apply to patients undergoing adjuvant oncological therapy [6]. Additionally, as many as 73% of patients strongly desire to have the ileostomy closure as soon as possible [7]. A study from Japan assessing very early ileostomy closure showed serious postoperative adverse events and was even terminated due to a high number of complications [8]. Other surgeons prefer to close ileostomies later, only after the oncological treatment is finished [9]. There is also concern about low anterior resection syndrome (LARS), which can be associated with the presence of a defunctioning loop ileostomy [10]. Systematic review and meta-analysis suggest that the prolonged time to ileostomy closure may negatively influence bowel function. Vogel et al. concluded that patients with prolonged preservation of defunctioning loop ileostomies have a significantly higher risk of major LARS [11]. Therefore, it is important to establish the optimal time for ileostomy closure in order to minimize its adverse effects. To date, no prospective assessment of Polish patients undergoing ileostomy closure has been performed; only small, preliminary studies have been conducted [12]. The objective of this study was to perform a comparison of early postoperative outcomes in early, standard, and delayed ileostomy reversal patients.

## 2. Materials and Methods 

The LIquidation of iLEOstomy (LILEO) study was a prospective multicenter cohort study that lasted from October 2022 until the end of December 2023 and focused on the clinical outcomes of patients undergoing ileostomy reversal (liquidation of the ileostomy procedure). A unified study protocol was sent to all participating surgical centers before the start of the study, providing precise instructions on inclusion and exclusion criteria, as well as on how the database should be filled in prospectively. Furthermore, the patient consent form and information about the study, provided in the form of a leaflet, were sent to each surgical center to ensure that every patient received the same information. The specially designed database included anonymous parameters divided into several categories: demographic data, ileostomy creation data (e.g., created by a specialist or resident, indications), perioperative care parameters, surgical procedure and technique data, complications, and postoperative outcomes (e.g., reoperations, mortality, and readmissions within 30 days of discharge). Out of 27 initially registered surgical centers in Poland, 19 centers provided data by the end of the study using a password-secured database.

The inclusion criteria consisted of the following requirements: patients undergoing ileostomy reversal who were older than 18 years of age and who provided informed consent to participate in the study. The exclusion criteria were the following: ileostomy reversal that occurred during a different procedure, for example during a hemihepatectomy, and concomitant parastomal hernia repair with mesh implantation. The data were obtained during the hospital stay and outpatient visits within 30 days after discharge. Ileostomy closure was possible after the assurance of healed rectal anastomosis. The healing was confirmed by endoscopy conducted prior to ileostomy closure.

**Figure 1:**
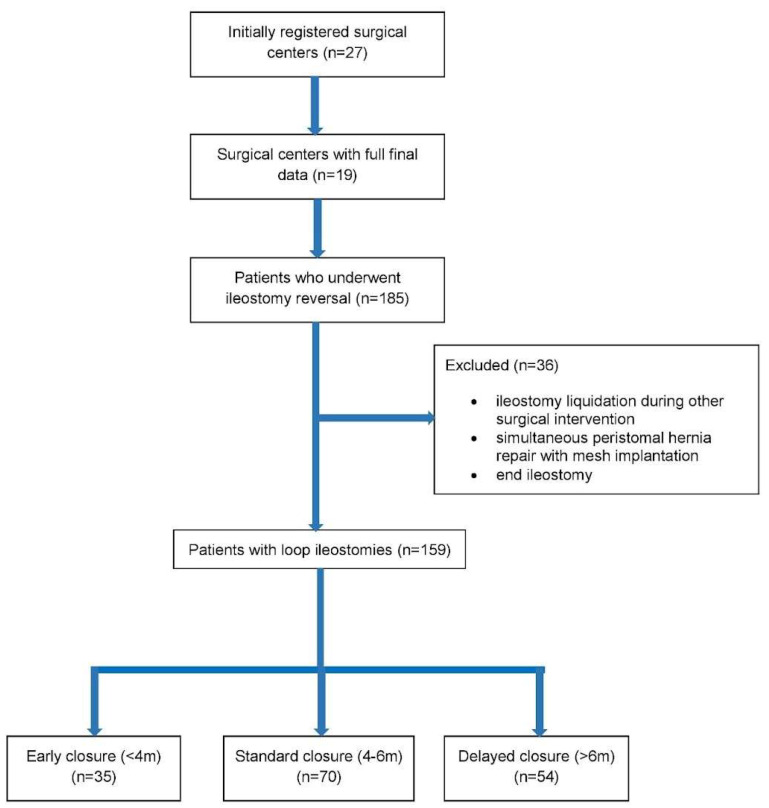
Flowchart of the study.

Information acquired from the surgical centers included data from 185 patients after ileostomy reversal and various demographic, clinical, and perioperative parameters. For this particular study, 159 patients who underwent loop ileostomy reversal were selected for further analysis (106 males, 53 females). Patients were divided into 3 groups according to the time of ileostomy reversal: (1) early (<4 months); (2) standard (4–6 months); and (3) delayed (>6 months). A flowchart showing the formation of the three study groups is presented below in Figure 1.

The primary goals of the study were the length of hospital stay (LOS), postoperative complications, and 30-day reoperation rate. The secondary goal was the assessment of the complication rate according to the Clavien–Dindo classification. Statistical analysis was performed using Statsoft STATISTICA v.13 (StatSoft Inc., Tulsa, OK, USA). Numerical variables are shown as mean and/or standard deviation (SD) with the use of median and interquartile range (IQR) when appropriate, and categorical variables are displayed using percentages. The Pearson chi-square test of independence was applied to examine the relationship between each variable and outcome. For normally distributed data, the Shapiro–Wilk test was used. In non-normally distributed quantitative variable groups, a comparison was made using the Kruskal–Wallis test. A *p*-value below 0.05 was considered statistically significant.

The study (KBKA/55/O/2022) was approved by the Bioethical Committee of the Andrzej Frycz-Modrzewski University in Cracow, Poland.

## 3. Results

The earliest ileostomy reversal took place 2 weeks after the initial ileostomy, and the latest occurred 28 months afterward. The median age at the time of ileostomy closure was 65 years in group 1, 64 years in group 2, and 66 years in group 3 (*p* = 0.635). The median body mass index (BMI) of the studied groups was 25.5, 26.2, and 26.4 kg/m^2^, respectively (*p* = 0.313). The number of female patients was higher in the early ileostomy reversal group, but, in reference to age, BMI, ASA scale, or comorbidities, such as ischemic heart disease, hypertension, and diabetes, no statistically significant differences between the groups were observed. The majority (>90%) of ileostomies were created by specialist surgeons in all three groups. Demographics and analysis of the study groups are presented in Table 1.

Operations were performed by specialist surgeons in 65.7% of surgeries in the early group, in 55.7% of surgeries in the standard group, and in 70.4% of surgeries in the delayed group; no statistically significant differences were seen (*p* = 0.45). Two cases of operator change from resident to specialist occurred; one in the standard reversal group and one in the delayed reversal group. The length of hospital stay (LOS) did not differ between the early, standard, and delayed ileostomy reversal groups. Median LOS was 5, 5.5, and 6 days in the three groups, respectively (*p* = 0.22). Postoperative complications were observed in all three ileostomy reversal groups: 34.3% in the early group, 24.3% in the standard group, and 33.3% in the delayed group (*p* = 0.43). No difference in the 30-day reoperation rate was observed between the early reversal (8.6%), standard reversal (2.9%), and delayed reversal groups (9.3%), (*p* = 0.28). The results according to the primary goals of the study are presented below in Table 2.

The wound infection rate in the early reversal group was 25.7%, it was 12.9% in the standard reversal group, and in the delayed group, it was the lowest at 7.4%. Despite the noticeable difference between the early reversal group (25.7%) and the delayed reversal group (7.4%), we observed no statistical significance (*p* = 0.05).

In a separate analysis of complications in the Clavien–Dindo scale with special consideration according to complication severity, we also did not notice a difference between light (grade 1 and 2) and serious (grade 3–5) complications (*p* = 0.95). The results according to the secondary goals of the study are presented below in Table 3.

## 4. Discussion

One of the novelties of our approach was to divide the study population into three groups of loop ileostomy reversal times: early, standard, and delayed. Most of the preceding studies were designed to compare very early versus delayed ileostomy reversal groups, or simply early versus delayed ileostomy reversal groups. In our multicenter prospective study, we also introduced a standard group, which, we believe, provides more clarity and answers the question regarding loop ileostomy closure timing that has been lingering for many years, with the first scientific inquiry occurring about 20 years ago [13].

Single-center studies performed previously were commonly retrospective in their character, and the results suggested better outcomes of early ileostomy closure [14,15]. Nevertheless, it is worth mentioning that those studies analyzed groups of patients operated on over a long time period, ranging from 5 to 12 years. During this time, perioperative care evolved significantly, due to the introduction of fast-track pathways, such as the ERAS protocol in colorectal surgery, which was introduced in 2005 [16,17]. Other retrospective studies by Zhou et al. and Sauri et al. failed to show any differences between early and delayed ileostomy reversal groups in terms of postoperative complications and LOS [18,19].

On the other hand, a prospective study with randomization from 3 Swiss hospitals was ended due to significantly increased postoperative complications in the early reversal group [20]. Similarly, a randomized study by Vogel et al., in which ileostomy reversal took place 7 to 12 days after proctocolectomy with ileal pouch construction, was also ended prematurely due to the unacceptably high number of postoperative complications in comparison to the delayed group [21]. However, the earlier closure of ileostomy in inflammatory bowel disease patients has been advocated by Morada et al. due to the risk of ileostomy site malignancies, which was found to be time-dependent; the longer the ileostomy was present, the higher was the risk of ileostomy site malignancy. Similarly, in patients with familial adenomatous polyposis, researchers believe that ileostomy reversal should be performed earlier [22,23].

Meta-analysis performed by Li Wang et al. found that early ileostomy closure can result in a greater number of postoperative complications with the predominance of wound infections. Conversely, delayed closure was associated with more preoperative stoma-related complications, such as skin irritation and stoma bag leakage. No difference was observed in the comparison of severe complications between groups [24]. Interestingly, the postoperative quality of life of ileostomy patients was similar when compared in the early and standard closure groups by the EORTC QLQ-C30 and LARS questionnaires [25].

One of the most common postoperative complications encountered by patients undergoing ileostomy closure is postoperative ileus (POI), accounting for as many as 20% of complications. The longer the ileostomy was present, the higher was the POI risk [26]. Also, the POI risk was higher in patients who had POI after initial colorectal surgery [27]. Loss of the microbiota in the malfunctioning bowel can be responsible for increased risk of perioperative complications. Probiotics were used preoperatively to minimize this, but eventually there was no difference in the final microbiota diversity observed in patients with and without complications [28]. Among the preoperative maneuvers that can potentially decrease the frequency of POI is bowel stimulation via the efferent limb of ileostomy [29]. Lloyd et al. performed a meta-analysis of available studies where the efferent limb of ileostomy was physiologically stimulated by administration of saline solution with a thickening agent that resulted in reduced postoperative complications and shortened LOS. This can be an additional factor favoring delayed ileostomy closure [30]. Also, postoperative gum chewing is a simple and effective way to prevent POI. The return of peristalsis was significantly faster when gum was chewed for 30 min approximately 6 h after surgery and repeated every 8 h, resulting in a shorter LOS [31].

Infections are also among the most common complications of ileostomy reversal. Surgical site infections (SSIs) occurred more frequently in patients undergoing early ileostomy reversal [19]. Many authors suggested that wound closure by purse-string sutures or by the use of incisional negative pressure wound therapy (iNPWT) may be a very effective way to prevent SSIs [32,33,34]. Moreover, *Clostridium difficile* infection is a postoperative complication affecting up to 4% of patients undergoing ileostomy reversal and, in a retrospective study by Richards et al., was shown to occur more often in the delayed ileostomy reversal groups [35,36]. The aforementioned arguments should be taken into account during the general assessment of prospective ileostomy reversal patients. Instead of solely using the time criterion (early, standard, or delayed), an individualized approach should be the basis for clinical decision making.

Lastly, many patients with defunctioning ileostomies undergo continuous systematic oncological therapy. Cheng et al. showed that ileostomy patients can undergo reversal surgery in adequate time following chemotherapy, without a consequent increase in postoperative complications. Nevertheless, patients receiving specific agents, such as bevacizumab, can develop major drug-specific complications, and special caution should be used when planning ileostomy reversals in such patients [37].

The limitations of our study include various surgical techniques and perioperative care practices and are due to data coming from different surgical departments across Poland. In this study, we did not analyze the methods of ileostomy closure and outpatient preoperative preparation for the procedure, which also could differ between surgical centers involved in this study. However, given the large number of patients analyzed prospectively, we are able to draw conclusions regarding the impact of ileostomy closure time on subsequent outcomes.

## 5. Conclusions

Our analysis showed that the time interval from index surgery until ileostomy reversal is not predictive for perioperative complications, for 30-day reoperation rate, or for LOS. When deciding on the optimal time for ileostomy reversal, many other clinical factors should be taken into consideration, including oncological prognosis and the current type of adjuvant therapy. Common postoperative complications, like POIs and SSIs, should be prevented by readily available evidence-based techniques, such as preoperative efferent-limb stimulation, postoperative gum chewing, purse-string suturing, or iNPWT.

## Figures and Tables

**Table 1:** The characteristics of the patients.

	**Early Closure (<4 m)**	**Standard Closure (4–6 m)**	**Delayed Closure (>6 m)**	***p***
Number of patients, n (%)	35	70	54	
**Demographics**				
Females, n (%)	19 (54.3%)	16 (22.9%)	18 (33.3%)	<0.05
Males, n (%)	16 (45.7%)	54 (77.1%)	36 (66.7%)	
Median age, (q1–q3)	65 (57–71)	64 (54–69)	66 (59–71)	0.635
Median BMI, kg/m^2^ (q1–q3)	25.5 (22.4–27.2)	26.4 (23–29.2)	26.2 (23.5–28.4)	0.313
ASA 1, n (%)	1 (2.9%)	6 (8.6%)	2 (3.7%)	0.55
ASA 2, n (%)	20 (57.1%)	44 (62.8%)	33 (61.1%)	
ASA 3, n (%)	14 (40%)	20 (28.6%)	19 (35.2%)	
Ischemic heart disease, n (%)	3 (8.6%)	6 (8.6%)	6 (11.1)	0.87
Hypertension, n (%)	17 (48.6%)	32 (45.7%)	23 (42.6%)	0.85
Diabetes, n (%)	4 (11.4%)	7 (10%)	8 (14.8%)	0.71
Ileostomy created by specialist, n (%)	32 (91.4%)	69 (98.6%)	52 (96.3%)	00.19
Ileostomy created by trainee, n (%)	3 (8.6%)	1 (1.4%)	2 (3.7%)	

**Table 2:** Outcomes of ileostomy reversal in the three groups according to the primary goals of the study.

Parameter Analyzed	Early	Standard	Delayed	*p*
Ileostomy reversal performed by specialist	23 (65.7%)	39 (55.7%)	38 (70.4%)	0.45
Ileostomy reversal performed by trainee	12 (34.3%)	30 (42.9%)	15 (27.8%)	
Ileostomy reversal started by trainee but converted to specialist	-	1 (1.4%)	1 (1.9%)	
Median LOS, IQD (days)	5 (4–8)	5,5 (4–6)	6 (5–8)	0.22
Complication	12 (34.3%)	17 (24.3%)	18 (33.3%)	0.43
30-day reoperation	3 (8.6%)	2 (2.9%)	5 (9.3%)	0.28

**Table 3:** Outcomes of ileostomy reversal in the three groups according to the secondary goals of the study.

Parameter Analyzed	Early	Standard	Delayed	*p*
Clavien–Dindo grade 1, n (%)	6 (17.1%)	6 (8.6%)	1 (1.9%)	0.07
Clavien–Dindo grade 2, n (%)	2 (5.7%)	5 (7.1%)	10 (18.5%)	
Clavien–Dindo grade 3, n (%)	3 (8.6%)	6 (8.6%)	6 (11.1%)	
Clavien–Dindo grade 4, n (%)	-	-	1 (1.9%)	
Clavien–Dindo grade 5, n (%)	1 (2.9%)	-	-	
Wound infection, n (%)	9 (25.7%)	9 (12.9%)	4 (7.4%)	0.05

## Data Availability

Data without personal patient information can be available upon request via email to the main author.
